# Epigenetic Differences in Cortical Neurons from a Pair of Monozygotic Twins Discordant for Alzheimer's Disease

**DOI:** 10.1371/journal.pone.0006617

**Published:** 2009-08-12

**Authors:** Diego Mastroeni, Ann McKee, Andrew Grover, Joseph Rogers, Paul D. Coleman

**Affiliations:** 1 L.J. Roberts Center for Alzheimer's Research, Sun Health Research Institute, Sun City, Arizona, United States of America; 2 Departments of Pathology and Neurology, Boston University School of Medicine, Boston, Massachusetts, United States of America; University of North Dakota, United States of America

## Abstract

DNA methylation [1], [2] is capable of modulating coordinate expression of large numbers of genes across many different pathways, and may therefore warrant investigation for their potential role between genes and disease phenotype. In a rare set of monozygotic twins discordant for Alzheimer's disease (AD), significantly reduced levels of DNA methylation were observed in temporal neocortex neuronal nuclei of the AD twin. These findings are consistent with the hypothesis that epigenetic mechanisms may mediate at the molecular level the effects of life events on AD risk, and provide, for the first time, a potential explanation for AD discordance despite genetic similarities.

## Introduction

Wide varieties of studies have examined candidate genes for associations with Alzheimer's disease (AD). Although such associations have been found, they are probabilistic rather than inevitable, with the exception of familial AD. A potential explanation of the probabilistic nature of the association between specific genes and disease may be the apparent genetic complexity of AD. However, the existence of rare monozygotic twins discordant for AD offers the opportunity to examine other factors that may be contributing to the probabilistic nature of the association between genes and AD, specifically epigenetic mechanisms. Since epigenetic modifications contribute to the phenotypic differences that emerge in monozygotic twins, including discordant disease states [3], we examined DNA methylation, an important epigenetic mechanism, in monozygotic twins discordant for Alzheimer's disease (AD). As has been described in sporadic AD cases [4], neurons of the AD twin exhibited dramatic decrements across multiple DNA methylation markers compared to the non-AD twin. Epigenetics may therefore, constitute a basic molecular genetic mechanism in the pathophysiology of AD.

## Methods

### Ethics Statement*

Written informed consent for autopsy was obtained for both cases in compliance with institutional guidelines of Boston University. The Boston University Institutional Review Board approved this study including recruitment, enrollment, and autopsy procedures. Both twins and their respective next-of-kin consented to brain autopsy for the purpose of research analysis as participants in the Boston University Alzheimer's Disease Center. The human brain tissue used in this manuscript was from routine existing autopsies, which fully qualifies for 4C exemption by NIH guidelines. In addition samples were analyzed anonymously (e.g. twin 1 and twin 2) throughout the experimental process.

### Subjects and brain samples

**Figure 1:**
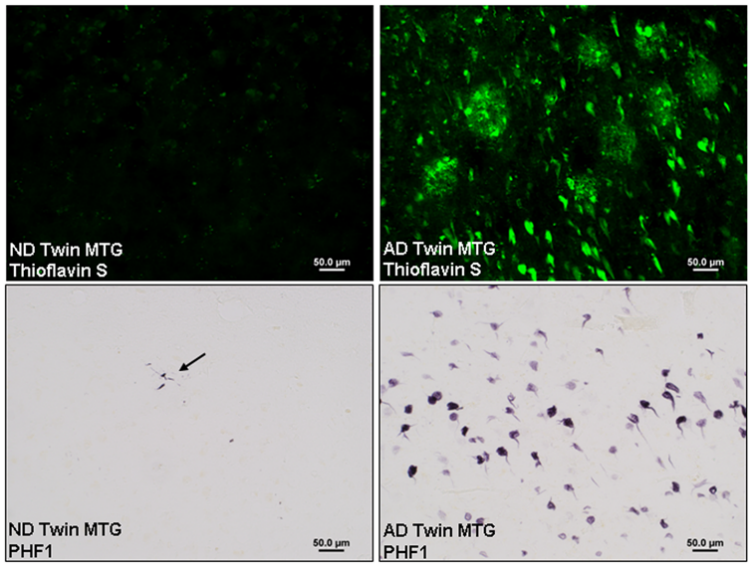
Thioflavin S plaques and PHF1 neurofibrillary tangle pathology in middle temporal gyrus (MTG) of non-demented and AD twin. AD pathology was remarkably rare in the non-demented twin and abundant in the AD twin.

Using standard protocols of NIH AD Centers, both twins were thoroughly evaluated antemortem and postmortem by board-certified neurologists and a neuropathologist who determined their respective diagnoses as AD and neurologically normal, non-demented (ND). The AD twin was a white male chemical engineer who had extensive pesticide contact in his work. He developed AD symptoms at age 60, first manifest as the inability to read maps, followed by progressive loss of memory and intellect over 16 years until his death at age 76. His identical twin, also a chemical engineer with an identical education but different work environment, died at 79 years from prostate cancer. At the time of his death, he was cognitively intact. The twins were autopsied at the same facility using the same tissue processing protocols. Post mortem delay for the control twin was 3 h 10 minutes and 7 h 20 minutes for the AD twin. Both subjects were immediately snap frozen on aluminum plates cooled to −80°C on dry ice and immediately transferred to −80°C freezer for long term storage. In the AD twin post-mortem examination confirmed severe AD (NIA-Reagan: high, CERAD plaque: frequent, Braak: VI). In the non-demented twin post-mortem examination revealed sparse neuritic plaques and entorhinal and transentorhinal NFTs (NIA-Reagan: low, CERAD: sparse, Braak: II). The AD twin showed dense plaques and neurofibrillary tangles (NFT) in the anterior temporal cortex, while these stigmata were remarkably rare in the non-demented twin (Figure 1).

### Immunohistochemistry

Temporal neo-cortex was sliced axially into 1-cm thick slabs, immersion fixed for 48 h in buffered 4% paraformaldehyde at 4°C, washed extensively in phosphate buffer (PB), and cryoprotected in ethylene glycol and glycerol. The slabs were then sectioned at 40 µm on a freezing cryostat. Free-floating sections were stored in freezing solution (glycol/glycerol/PB) at −20°C until required for experiments. Tissue sections used for bright field microscopy were immunoreacted using the avidin-biotin complex/diaminobenzidine (DAB) method. Briefly, tissues were washed 2×, blocked in 1% hydrogen peroxide for 45 min, washed 3×, blocked in 3% bovine serum albumin (BSA) for 1 h, washed 2×, and incubated at 4°C overnight in primary antibody solutions containing 0.25% BSA. Unless otherwise stated, all washes were with 1× PBS Triton (PBST). Available information about the antibodies is given in (Table 1). After incubation with primary antibody, sections were washed 3×, incubated in biotinylated, species-specific secondary antibodies (Vector) for 2 h, washed 3×, and incubated in avidin-biotin complex (Pierce) for 1 h. Following incubation with secondary antibody, the sections were washed 3×, once in PBST and twice in 0.05 M Tris buffer, then exposed to DAB solution containing 125 µl of 5 mg/ml DAB (Sigma), 11.125 ml 50 mM Tris buffer pH 7.6, and 500 µl saturated nickel ammonium sulfate. Incubations during chromagen development were no longer than 10 min, and were followed by two quick rinses in 50 mM Tris to stop the reaction. Finally, the sections were dried, taken through graded alcohols, de-fatted in Neoclear (EMD), and mounted with Permount (Pierce). AD and ND sections were immunoreacted simultaneously using netwells in well-less plates. For fluorescence microscopy, the sections were washed 3×, blocked with either 3% normal goat serum or 3% BSA, and incubated for 2 h. The sections were then washed 2×, incubated in primary antibody in 0.25% BSA at 4°C overnight, washed again, and incubated in species–specific, fluorophore-conjugated secondary antibodies (Molecular Probes) at room temperature for 2 h. After a final wash, the sections were mounted, taken through Sudan Black to reduce autofluorescence, and coverslipped with Vectashield mounting media (Vector). Deletion of primary antibody or incubation with pre-immune serum resulted in abolition of specific immunoreactivity in all cases (data not shown). Adjacent serial sections were stained with cresyl violet for cell layer identification and verification that the island neurons of layer II were intact. For some sections, nuclei were counterstained with 4′,6′-diamidino-2-phenylindole (DAPI) (Invitrogen) before mounting.

**Table 1:** Antibodies.

Antibody	Host	Dilution	Source/Catalogue#	Recognition/sequence	References
NSE	Chicken plyclonal	1∶500	Chemicon/AB9698	Neuron Specific Enolase	www.millipore.com
GFAP	Rabbit polyclonal	1∶1000	Chemicon/ab5804	Glial fibrillary acidic protein is a class-III intermediate filament	[17]
RAC1	lectin	1∶1000	Vector/B-1085	Ricinus communis agglutinin I	[18]
pS396	Rabbit polyclonal	1∶1000	Invitrogen/44-752G	Serine 396	[19]
PHF1	Mouse monoclonal	1∶1000	Gift of Dr. P. Davies	Paired Helical Filament 1	[20]
MBD3	Mouse monoclonal	1∶400	Abcam/ab45027	CKAFMVTDEDIRKQEE	[21]
5-methylcytidine	Mouse monoclonal	1∶1000	Genway/20-783-71663	Methylation	[22]
MTA2	Rabbit polyclonal	1∶500	Abcam/ab8106	Amino acids 652–668	[23]
HDAC2	Rabbit monoclonal	1∶500	Abcam/ab32117	Reidues within C-terminal end	[24]
5-methylcytosine	Mouse Monoclonal	1∶1000	Genway/20-003-40005	Methylation	[25]

Double-label immunohistochemistry was also employed to evaluate the associations of epigenetic factors with specific neuronal, and glial cell types. Briefly, sections were washed, blocked with either 3% normal goat serum or 3% BSA, and incubated for 1–2 h. Sections were then washed 2×, incubated in primary antibodies raised in different species in 0.25% BSA/PBST at 4°C overnight. After primary incubation sections were washed 3× in PBST, and incubated in species–specific, fluorophore-conjugated secondary antibodies (Molecular Probes) at room temperature for 2 h. After a final wash, the sections were mounted, taken through Sudan Black to reduce autofluorescence, and coverslipped with Vectashield mounting media (Vector). Immunostained tissue sections were examined on Olympus IX51 and Olympus IX70 microscopes equipped with epifluorescence illumination or with confocal laser scanning using argon and krypton lasers (Olympus IX70). The findings were documented photographically with an Olympus DP-71 color digital camera or, for confocal microscopy, by Fluoview software (Olympus).

### Cell Quantification and statistical analysis

Using the Fluoview software, integrated fluorescence intensities for 5-methylcytosine of layer II and layer III of the anterior temporal neocortex from each individual were analyzed. Briefly, sections were processed in parallel using identical antibody concentrations. DAPI+Nuclei of NSE+ cells were randomly selected in coded slides. The detection bandwidth was set at 488 nm for 5-methylcytosine indicator, and fluorescence intensity of the nucleus was recorded (N = 20 per layer per case).

## Results

### DNA methylation Immunoreactivity

**Figure 2:**
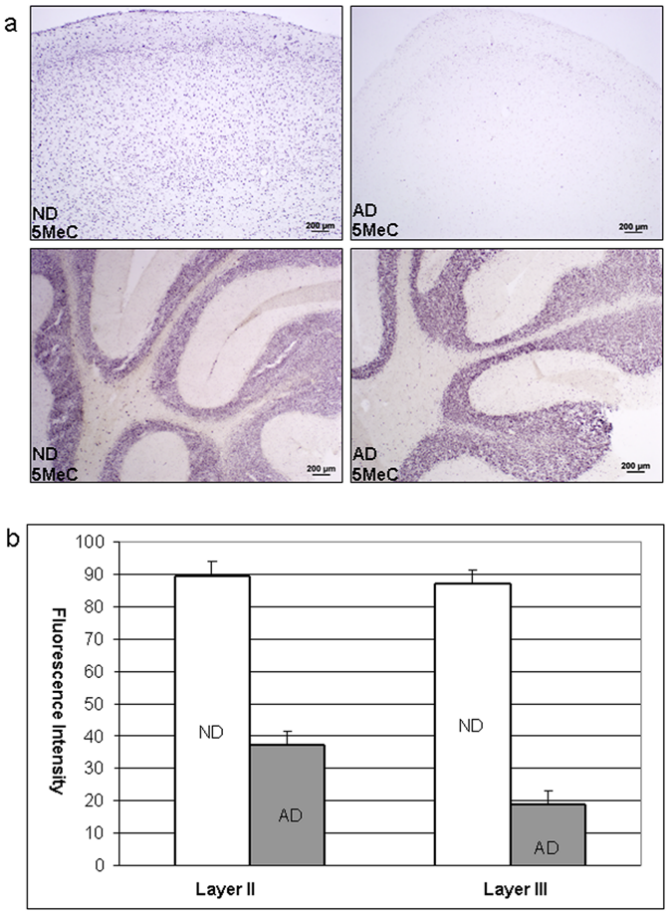
a) Anterior temporal neocortex (left and right top panels) and cerebellum (left and right bottom panels) immunoreactivity for DNA methylation marker in a pair of monozygotic twins discordant for Alzheimer's disease. Sections were processed in parallel using identical antibody concentrations to 5-methylcytosine (1∶1000) with nickel intensification. Note similarity of immunoreactivity in cerebellar granule cell layer of both twins in this brain region that is relatively unaffected in Alzheimer's disease. b) Integrated fluorescence intensities of DAPI-counterstained anterior temporal neocortex layer II and layer III neuronal nuclei in a set of monozygotic twins discordant for AD. Sections were processed at the same time using identical immunohistochemical methods [4] and antibody concentrations. Nuclei of NSE+ cells (N = 20/brain) were randomly selected and traced from coded slides by an investigator blind to subject condition. The appropriate detection fluorescence bandwidth (488 nm) for 5-methylcytosine indicator was then set, and the integrated fluorescence intensity within the traced area was taken. Highly significant decrements in the pathologically-vulnerable entorhinal cortex was observed in the AD twin (p<3.51E-08 for layer II and p<3.71E-06 for layer III), whereas readings for the cerebellum were nearly identical in the AD and ND twin (data not shown).

Figure 2a shows representative immunohistochemistry of anterior temporal neocortex for 5-methylcytosine, a marker of methylated CpG sites on DNA. In the AD twin, decreased immunoreactivity relative to the ND twin was readily apparent in the anterior temporal neocortex, a region severely affected in AD. Similar results were obtained in the pathologically-vulnerable superior frontal gyrus (Figure S1). To develop quantitative data, integrated fluorescence intensities of immunoreactivity for 5-methylcytosine were recorded for layer II and layer III nuclei of DAPI-and neuron specific enolase positive cells from coded slides. By two-tailed t-test, all markers exhibited highly significant (P<0.0001) decrements in immunoreactivity in the AD twin compared to the ND twin (Figure 2b). These findings are consistent with our previous results for non-twin AD and ND subjects [4].

To show that the results obtained in neocortex were not due to differences in tissue handling, storage time, or quality, DNA methylation markers were also evaluated in cerebellum, a brain region that is largely spared from AD pathology. As in our previous assessment of AD and control cases [4], the cerebella of both twins exhibited virtually identical staining patterns and intensity for 5-methylcytosine (Figure 2a), as well as for another methylation marker, 5-methylcytidine (Figure S1) and several methylation stabilizing factors (MBD2/3 and HDAC2; Figure S2)

### Relationship of DNA methylation marker 5-metylcytosine to neurons and Glial cells

**Figure 3:**
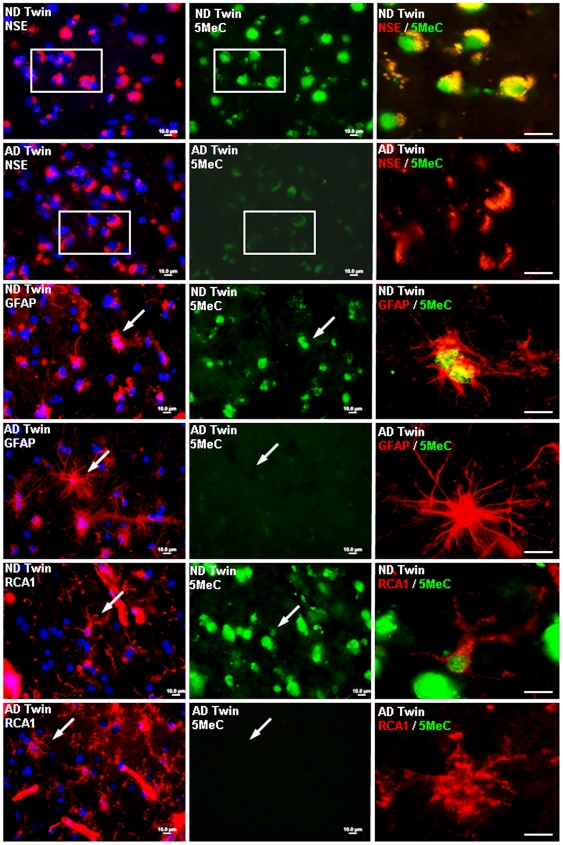
Co-localization of immunoreactivity for 5-methylcytosine with neuron specific enolase, GFAP, RCA1 (microglial marker) in non-demented and Alzheimer's disease twin. Note extensive co-localization of 5-methylcytosine in ND twin with all three cell specific markers, and the lack of co-localization with 5-methylcytosine in the AD twin.

In Figure 3 we examine co-localization of the DNA methylation marker, 5-methylcytosine, with markers for neurons (neuron specific enolase), for reactive astrocytes (GFAP) and for microglia (RCA1). These figures show that DNA methylation is present in all three cell types in non-demented brain and that the decrement in DNA methylation seen in AD extends to all three cell types.

## Discussion

Large-scale expression array studies have reported significant up- or downregulation of thousands of genes in AD [5], [6]. These alterations in gene expression span multiple pathogenic pathways, including amyloid β peptide (Aβ) processing, tau hyperphosphorylation, and inflammation, among others. Because many genes across the genome have methylation sites in their promoters [2], extensive hypomethylation in AD may provide an over-arching principle that accounts for significant aspects of the molecular and pathogenic complexity of the disorder. For example, amyloid precursor protein (APP), the Aβ precursor, has been shown to be normally methylated, and hypomethylated with age [7] which apparently enhances Aβ production [8]. However, more recent data indicates no difference in methylation of the APP gene in AD [9]. Furthermore, inducible nitric oxide synthase, interleukin 1, and tumor necrosis factor-α are all increased as part of the inflammatory response in AD cortex [10]; all their respective genes are methylated; and all show enhanced secretion with hypomethylation [11], [12], [13]. At the protein level, protein phosphatase 2A is methylated (probably by peptidylarginine methyltransferases or lysine methyltransferases), and its hypomethylation results in tau hyperphosphorylation [14].

The present findings indicate that epigenetic mechanisms may provide a molecular basis for the effect of life events, including exposure to hazardous substances, on AD risk. More specifically, they may provide a rationale for the consistent epidemiologic and neuropathologic association of AD with homocysteine elevation and folate deficiencies [15], [16], since folate ultimately provides the methyl group for DNA methylation. Maintaining adequate dietary folate (and B_12_) or increasing S-adenosylmethionine levels might therefore be useful, inexpensive strategies to decrease risk for AD. Since epigenetic patterns can be passed on to subsequent generations, epigenetics may also constitute a mechanism by which AD in a first degree relative confers increased risk of “sporadic” disease.

## Supporting Information

Figure S1 Top four panels: Immunoreactivity for 5-methylcytodine (5MeCd), another methylation marker, in medial temporal gyrus and cerebellum of non-demented and Alzheimer's disease twin. Note similarity of immunoreactivity in granule cell layer of both twins in cerebellum, which is relatively unaffected in Alzheimer's disease. Bottom two panels: 5-methylcytosine (5MeC)immunoreactivity in the Superior frontal gyrus (SFG)in AD and non-demented twin.
(2.63 MB TIF)

Figure S2 Immunoreactivity for selected components of the MECP1 complex in superior frontal gyrus and cerebellum of non-demented and AD twin. Note consistency of these results with other data shown.
(1.63 MB TIF)

## References

[1] Adcock IM, Tsaprouni L, Bhavsar P, Ito K (2007). Epigenetic regulation of airway inflammation.. Curr Opin Immunol.

[2] Suzuki MM, Bird A (2008). DNA methylation landscapes: provocative insights from epigenomics.. Nat Rev Genet.

[3] Poulsen P, Esteller M, Vaag A, Fraga MF (2007). The epigenetic basis of twin discordance in age-related diseases.. Pediatr Res.

[4] Mastroeni D, Grover A, Delvaux E, Whiteside C, Coleman PD (2008). Epigenetic changes in Alzheimer's disease: Decrements in DNA methylation.. Neurobiol Aging.

[5] Liang WS, Dunckley T, Beach TG, Grover A, Mastroeni D (2008). Altered neuronal gene expression in brain regions differentially affected by Alzheimer's disease: a reference data set.. Physiol Genomics.

[6] Liang WS, Reiman EM, Valla J, Dunckley T, Beach TG (2008). Alzheimer's disease is associated with reduced expression of energy metabolism genes in posterior cingulate neurons.. Proc Natl Acad Sci U S A.

[7] Tohgi H, Utsugisawa K, Nagane Y, Yoshimura M, Genda Y (1999). Reduction with age in methylcytosine in the promoter region −224 approximately −101 of the amyloid precursor protein gene in autopsy human cortex.. Brain Res Mol Brain Res.

[8] West RL, Lee JM, Maroun LE (1995). Hypomethylation of the amyloid precursor protein gene in the brain of an Alzheimer's disease patient.. J Mol Neurosci.

[9] Wang SC, Oelze B, Schumacher A (2008). Age-specific epigenetic drift in late-onset Alzheimer's disease.. PLoS ONE.

[10] Akiyama H, Barger S, Barnum S, Bradt B, Bauer J (2000). Inflammation and Alzheimer's disease.. Neurobiol Aging.

[11] Chan GC, Fish JE, Mawji IA, Leung DD, Rachlis AC (2005). Epigenetic basis for the transcriptional hyporesponsiveness of the human inducible nitric oxide synthase gene in vascular endothelial cells.. J Immunol.

[12] Kovacs EJ, Oppenheim JJ, Carter DB, Young HA (1987). Enhanced interleukin-1 production by human monocyte cell lines following treatment with 5-azacytidine.. J Leukoc Biol.

[13] Sato TA, Mitchell MD (2006). Molecular inhibition of histone deacetylation results in major enhancement of the production of IL-1beta in response to LPS.. Am J Physiol Endocrinol Metab.

[14] Sontag E, Nunbhakdi-Craig V, Sontag JM, Diaz-Arrastia R, Ogris E (2007). Protein phosphatase 2A methyltransferase links homocysteine metabolism with tau and amyloid precursor protein regulation.. J Neurosci.

[15] Seshadri S, Beiser A, Selhub J, Jacques PF, Rosenberg IH (2002). Plasma homocysteine as a risk factor for dementia and Alzheimer's disease.. N Engl J Med.

[16] Luchsinger JA, Patel B, Tang MX, Schupf N, Mayeux R (2008). Body mass index, dementia, and mortality in the elderly.. J Nutr Health Aging.

[17] Jungling K, Nagler K, Pfrieger FW, Gottmann K (2003). Purification of embryonic stem cell-derived neurons by immunoisolation.. Faseb J.

[18] Mannoji H, Yeger H, Becker LE (1986). A specific histochemical marker (lectin Ricinus communis agglutinin-1) for normal human microglia, and application to routine histopathology.. Acta Neuropathol.

[19] Alonso AD, Zaidi T, Novak M, Barra HS, Grundke-Iqbal I (2001). Interaction of tau isoforms with Alzheimer's disease abnormally hyperphosphorylated tau and in vitro phosphorylation into the disease-like protein.. J Biol Chem.

[20] Chapman G, Beaman BL, Loeffler DA, Camp DM, Domino EF (2003). In situ hybridization for detection of nocardial 16S rRNA: reactivity within intracellular inclusions in experimentally infected cynomolgus monkeys–and in Lewy body-containing human brain specimens.. Exp Neurol.

[21] Boyes J, Bird A (1991). DNA methylation inhibits transcription indirectly via a methyl-CpG binding protein.. Cell.

[22] Espada J, Ballestar E, Fraga MF, Villar-Garea A, Juarranz A (2004). Human DNA methyltransferase 1 is required for maintenance of the histone H3 modification pattern.. J Biol Chem.

[23] Caballero R, Setien F, Lopez-Serra L, Boix-Chornet M, Fraga MF (2007). Combinatorial effects of splice variants modulate function of Aiolos.. J Cell Sci.

[24] Weichert W, Roske A, Niesporek S, Noske A, Buckendahl AC (2008). Class I histone deacetylase expression has independent prognostic impact in human colorectal cancer: specific role of class I histone deacetylases in vitro and in vivo.. Clin Cancer Res.

[25] Maki WC, Mishra NN, Cameron EG, Filanoski B, Rastogi SK (2008). Nanowire-transistor based ultra-sensitive DNA methylation detection.. Biosens Bioelectron.

