# Supplementary figures and images for: Epigenetic Differences in Cortical Neurons from a Pair of Monozygotic Twins Discordant for Alzheimer's Disease
Source: PLoS One. 2009 Aug 12;4(8):e6617. doi: 10.1371/journal.pone.0006617 (PMC2719870; doi:10.1371/journal.pone.0006617)

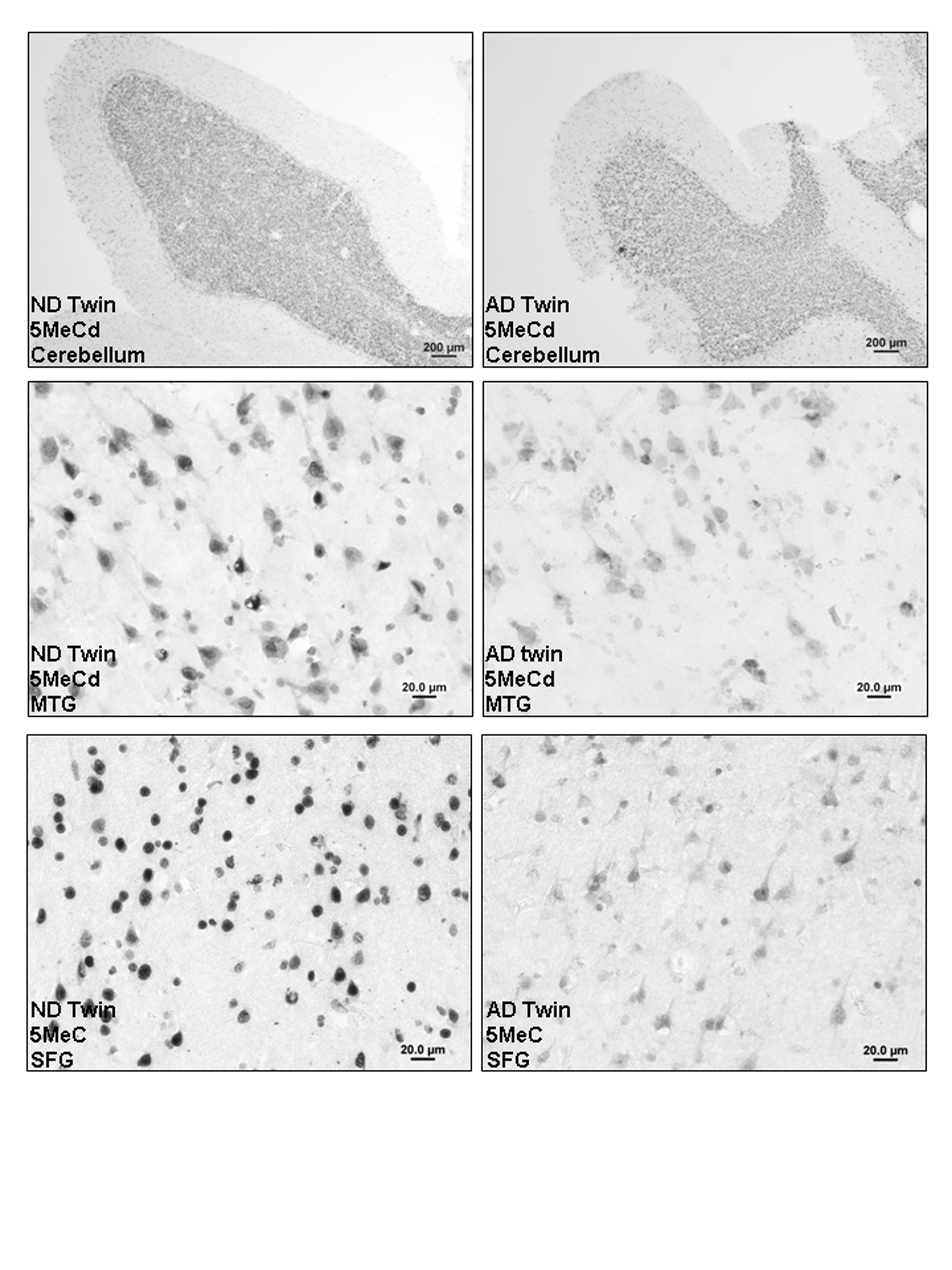

Supplement: Figure S1 — Top four panels: Immunoreactivity for 5-methylcytodine (5MeCd), another methylation marker, in medial temporal gyrus and cerebellum of non-demented and Alzheimer's disease twin. Note similarity of immunoreactivity in granule cell layer of both twins in cerebellum, which is relatively unaffected in Alzheimer's disease. Bottom two panels: 5-methylcytosine (5MeC)immunoreactivity in the Superior frontal gyrus (SFG)in AD and non-demented twin. (2.63 MB TIF) [file pone.0006617.s001.tif]

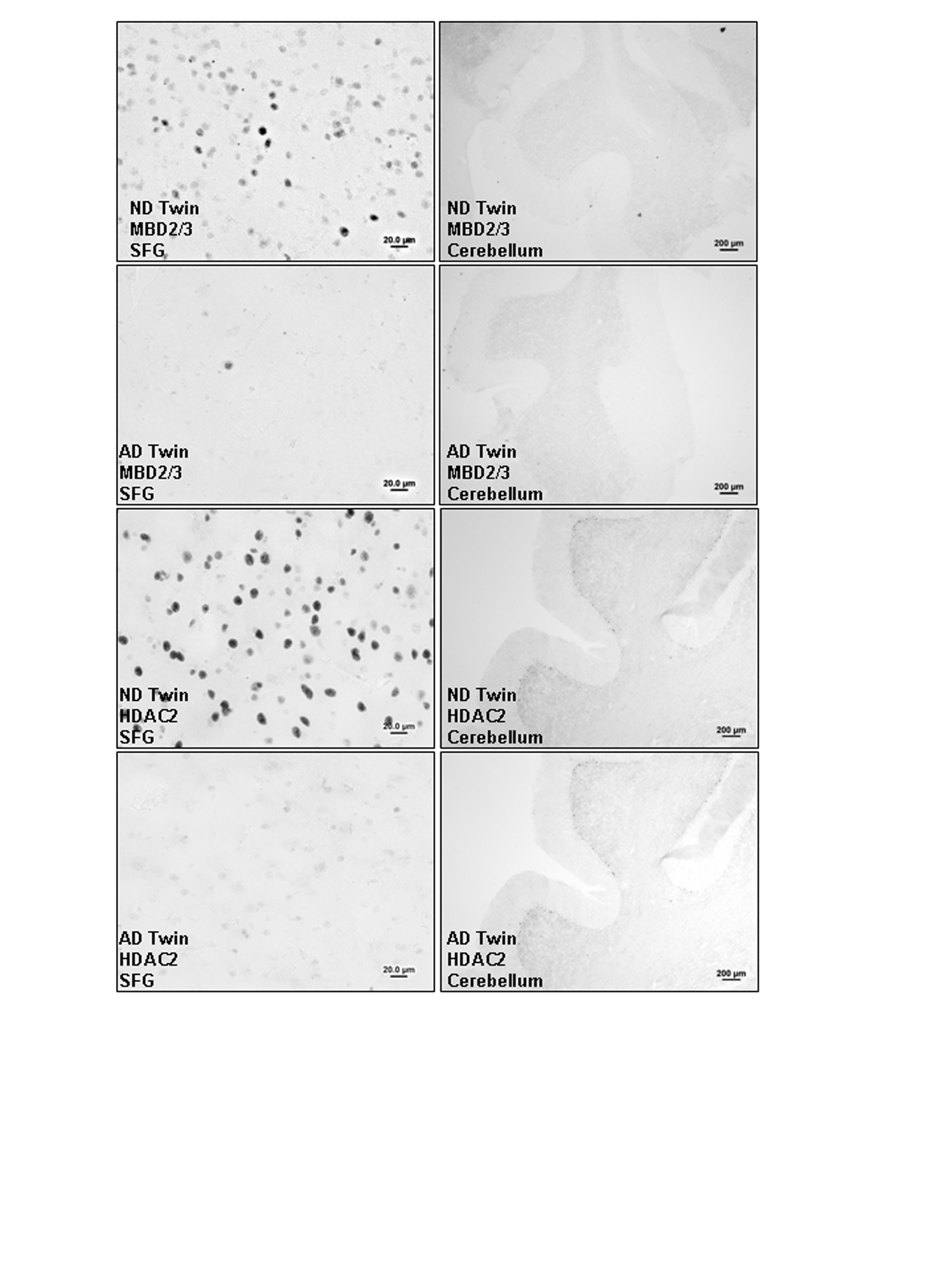

Supplement: Figure S2 — Immunoreactivity for selected components of the MECP1 complex in superior frontal gyrus and cerebellum of non-demented and AD twin. Note consistency of these results with other data shown. (1.63 MB TIF) [file pone.0006617.s002.tif]
